# Supplementary material for: Exogenous Application of Polycationic Nanobactericide on Tomato Plants Reduces the Candidatus Liberibacter Solanacearum Infection
Source: Plants (Basel). 2021 Oct 3;10(10):2096. doi: 10.3390/plants10102096 (PMC8539364; doi:10.3390/plants10102096)
Supplement: Supplementary file 1 [file plants-10-02096-s001.zip › plants-1397926-supplementary.pdf]

Supplementary materials

Figure S1 shows the ^{1}H NMR analysis of the isolated copolymer revealed the presence of ω-RAFT end groups (δ= 7.83, 7,45 and 7.30 ppm) and the characteristic signals derived from DMAEMA (δ= 1.2 (-C-CH_3), 2.6 (-CH_2-N), 2.2 (-N-$(CH_3)_2$), and 3.9-4.1 ppm (-O-CH_2)) and BMA (δ= 0.85 (-CH_2-CH_3), and 3.8-3.9 ppm (-O-CH_2)). The values of integrals of the -O-CH2 (i,c) groups of both co-monomers allowed to calculate the copolymer composition, which corresponds to 29 % of BMA units.

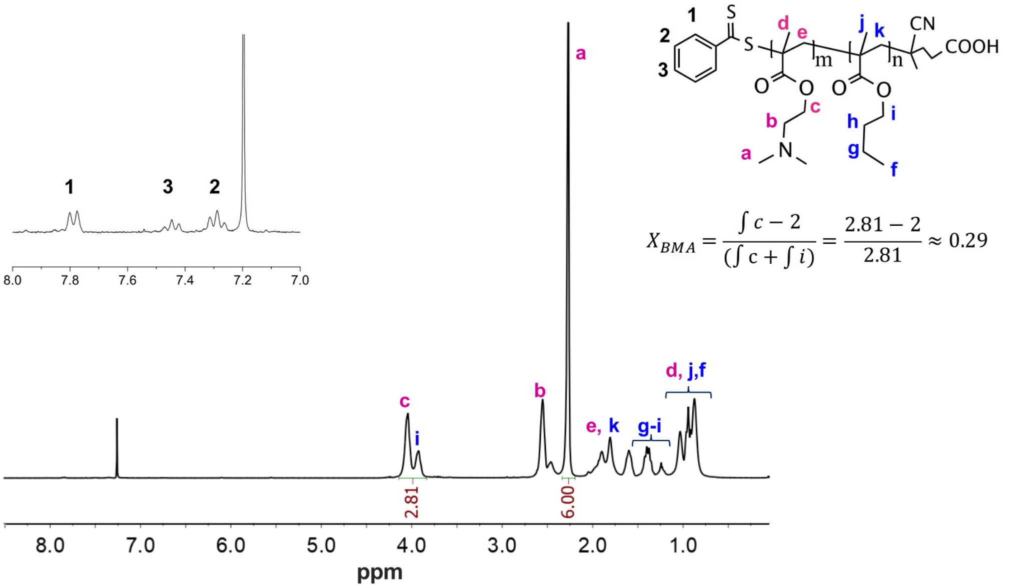

Figure S1. 1H NMR spectrum of P(DMAEMA-*co*-BMA), recorded in CDCl3, with peak assignments. Inset is an expanded region highlighting the presence of the ω-RAFT end group protons.

The signal e′ δ= 2.21 ppm represents the six protons of the methyl group bound to nitrogen. Their intensity decreases progressively after the partial alkylation with methyl iodide generating a signal displaces at 3.14 ppm (e), and that corresponds to the nine protons of the quaternary amino group.

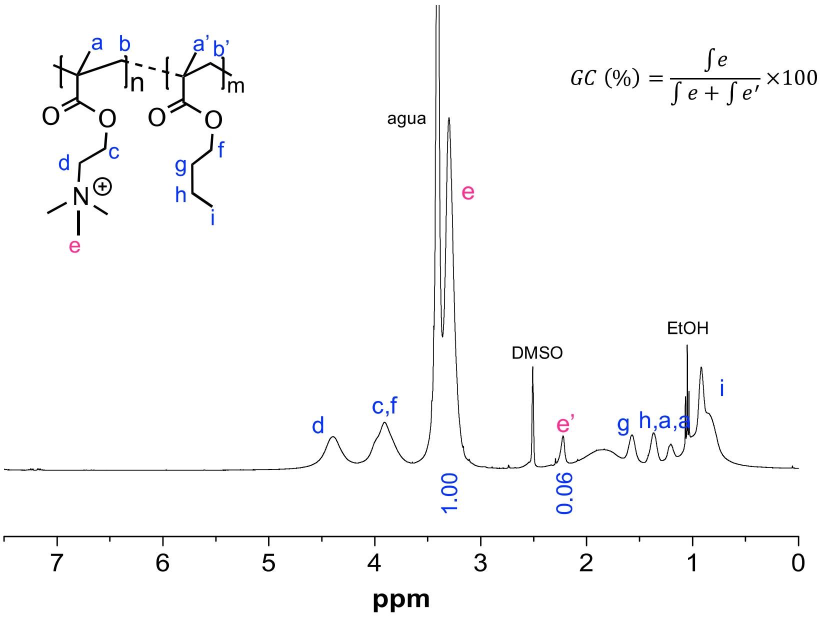

Figure S2. 1H NMR spectrum of P(METAI-*co*-BMA), recorded in DMSO-d6.

PNB **Control**

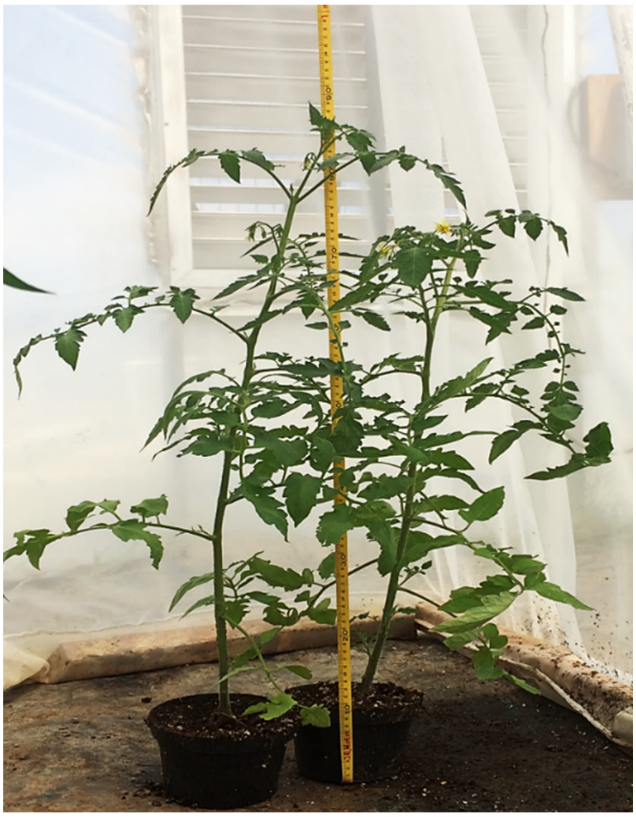

Figure S3. Healthy plants of tomato (*Solanum lycopersicum*) at 21 days of treatment with 70 ppm of PNB (**PNB**) and water treated (**Control**). Plants were grown in greenhouse conditions in absence of *Bactericera cockerelli* and *Candidatus* Liberibacter solanacearum.

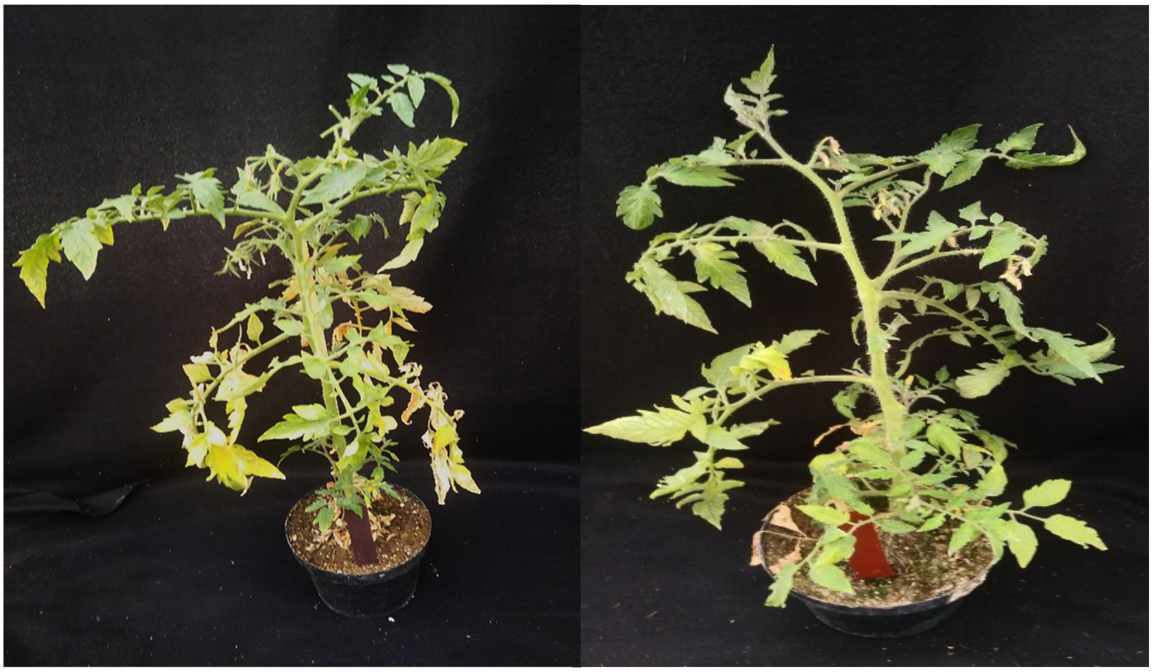

0 ppm **70 ppm**

Figure S4. Infected plants of tomato (*Solanum lycopersicum*) with *Candidatus* Liberibacter solanacearum (*Ca*Lso) at 7 days of treatment with 70 ppm of PNB.

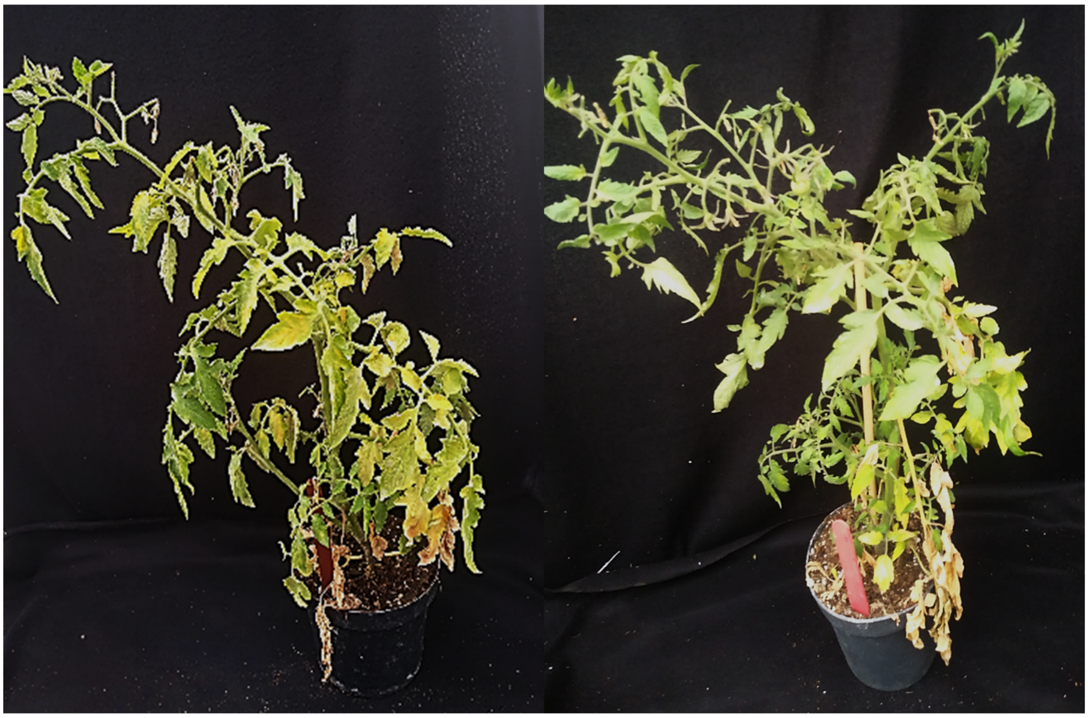

0 ppm **70 ppm**

Figure S5. Infected plants of tomato (*Solanum lycopersicum*) with *Candidatus* Liberibacter solanacearum (*Ca*Lso) at 14 days of treatment with 70 ppm of PNB.

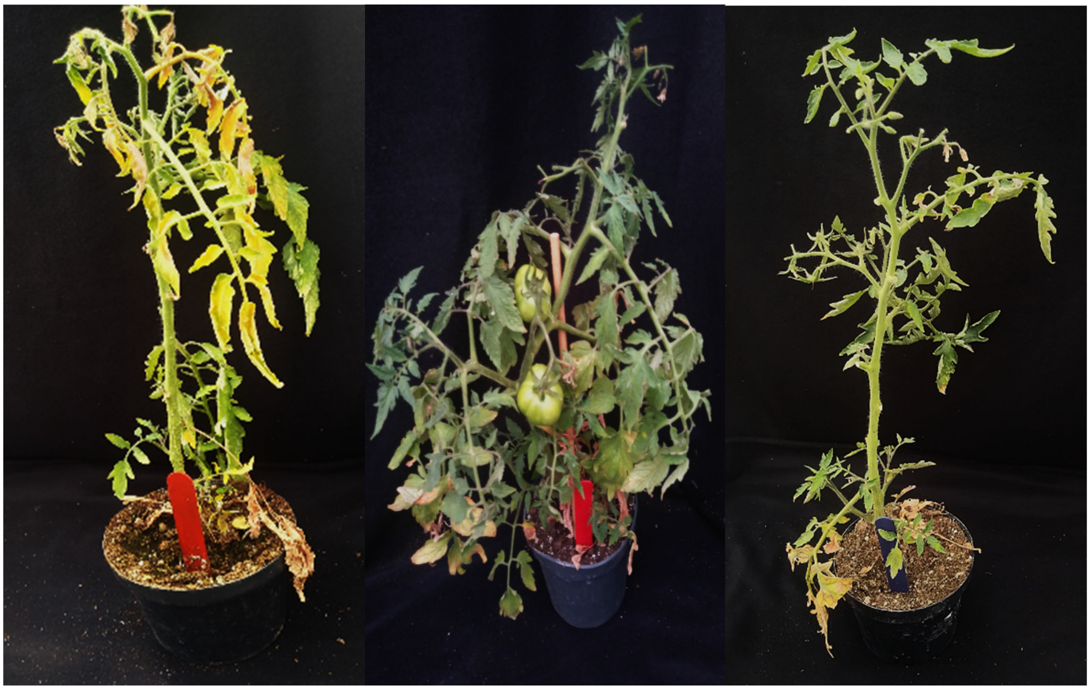

0 ppm **70 ppm** **OXY**

Figure S6. Infected plants of tomato (*Solanum lycopersicum*) with *Candidatus* Liberibacter solanacearum (*Ca*Lso) at 21 days of treatment with 70 ppm of PNB and oxytetracycline.
